# Synergy between HDAC and PARP Inhibitors on Proliferation of a Human Anaplastic Thyroid Cancer-Derived Cell Line

**DOI:** 10.1155/2015/978371

**Published:** 2015-01-29

**Authors:** Federica Baldan, Catia Mio, Lorenzo Allegri, Cinzia Puppin, Diego Russo, Sebastiano Filetti, Giuseppe Damante

**Affiliations:** ^1^Department of Medical and Biological Sciences, University of Udine, Piazzale Kolbe 4, 33100 Udine, Italy; ^2^Department of Health Sciences, University “Magna Graecia” of Catanzaro, 88100 Catanzaro, Italy; ^3^Department of Internal Medicine and Medical Specialties, University of Roma “La Sapienza”, 00198 Rome, Italy; ^4^Institute of Medical Genetics, University Hospital “S. Maria della Misericordia”, 33100 Udine, Italy

## Abstract

Anaplastic thyroid carcinoma (ATC) is a very aggressive human malignancy, having a marked degree of invasiveness and no features of thyroid differentiation. It is known that either HDAC inhibitors or PARP inhibitors have antiproliferative effects on thyroid cancer cells. Therefore, in this study the possible synergy between the two types of compounds has been investigated. The ATC-derived cell line SW1736 has been treated with the HDAC inhibitor suberoylanilide hydroxamic acid (SAHA) and the PARP inhibitor PJ34, alone or in combination. In terms of cell viability, the combination index value was always lower than 1 at various tested dosages, indicating, therefore, synergy in a wide range of doses for both compounds. Synergy was also observed in induction of apoptosis. In terms of thyroid-specific gene expression, synergy was observed for TSHR mRNA levels but not for NIS, TTF1, TTF2, and PAX8 mRNA levels. Altogether, these data suggest that the combined use of HDAC and PARP inhibitors may be a useful strategy for treatment of ATC.

## 1. Introduction

Thyroid cancer is the most common endocrine malignancy, and its incidence has continuously increased in the last three decades all over the world [1]. Thyroid cancers are typically classified as papillary (PTC), follicular (FTC), medullary (MTC), or anaplastic (ATC) carcinomas.

ATC is one of the most aggressive human malignancies. These tumors have a marked degree of invasiveness and extensive necrosis and there are no features of thyroid differentiation [2]. The mechanisms underlying the development of ATCs are incompletely understood. Currently, available therapy for ATCs includes chemotherapy, radiotherapy, and surgery [3]. Nonetheless, patients with ATC still have a median survival of 5 months and less than 20% survive 1 year. Furthermore early tumor dissemination results in 20–50% percent of patients having distant metastases and 90% having adjacent tissue invasion on presentation [2].

HDAC inhibitors (HDACIs) are a group of small molecules that promote gene transcription by chromatin remodeling and have been extensively studied as potential drugs for treating cancer. Luong et al. have established that the HDAC inhibitor suberoylanilide hydroxamic acid (SAHA), already FDA-approved for the treatment of several neoplastic diseases [4, 5], has antitumor activities against thyroid cancer [6].

Inhibitors of the poly(ADP-ribose) polymerases (PARPs) family are currently being evaluated as potential anticancer drugs. PARPs have a key role in a large number of cell viability processes as DNA repair, genome integrity, regulation of transcription, proliferation, and apoptosis [7].

Different independent studies have demonstrated that the combination of both HDAC inhibitors and PARP inhibitors with other drugs could result in synergistic effects on their antitumor activities if compared to those observed using single agents [8, 9].

Current cancer therapy should satisfy requirements for targeted elimination of cancer cells simultaneously with life-compatible adverse effects [10]. One of the main tenets of cancer therapeutics is that combinations of anticancer agents with different targets or different mechanisms of action and varied normal tissue toxicities will produce better therapeutic outcomes [11] by decreasing single drugs doses and minimizing or slowing drug resistance development. In this study, we investigated the possible use of SAHA, an HDAC inhibitor, and PJ34, a PARP inhibitor, in combination, in a cellular model of anaplastic thyroid cancer.

## 2. Material and Methods

### 2.1. Cell Line and Treatments

SW1736, human cell line derived from anaplastic thyroid cancer, was grown in RPMI 1640 medium (EuroClone, Milan, Italy) supplemented with 10% fetal bovine serum (Gibco Invitrogen, Milan, Italy) and 50 mg/mL gentamicin (Gibco Invitrogen, Milan, Italy) in a humidified incubator (5% CO_2_ in air at 37°C). The identity of SW1736 cells was demonstrated by evaluating the following STRs: D16S539, THO1, vWA, D3S1358, D21S11, and D18S51; the obtained genotype was identical to those reported by the CLS Cell Lines Service GmbH (http://www.cell-lines-service.de/). Cultured cells were treated with the following agents, either alone or in combination, as described in the text: SAHA (1–4 *μ*M in DMSO) (Cayman Chemical, Michigan, USA) and PJ34 (5–30 *μ*M in nuclease-free water) (Merck Chemicals Ltd). These concentrations are consistent with those utilized for* in vivo* studies [12, 13]. All treatments were done for 72 hours.

### 2.2. Cell Viability

To test cell viability, CellTiter-Blue Cell Viability assay (Promega, Milano, Italy) was used according to the manufacturer's instructions. Cells were seeded onto 96-well plates in 200 *μ*L medium. The next day, the growth medium was replaced with fresh medium containing DMSO as vehicle (untreated cultures) or SAHA and PJ34 alone or in combination. For each treatment quadruplicate wells were used.

### 2.3. Combination Index (CI Value)

Effects of drugs combination used in this study were evaluated using the combination index equation based on the multiple drug-effect equation of Chou-Talalay [14, 15]. In all cases where CI value could be determined the following diagnostic rule was applied: CI < 1 indicates synergism, CI = 1 indicates additive effect, and CI > 1 indicates antagonism. The analysis was obtained on CompuSyn software (ComboSyn Inc., Paramus, USA).

### 2.4. Annexin V Staining

Cells were treated with appropriate drugs as described and then they were washed with cold PBS, transferred to a polystyrene round-bottomed flow tube (Falcon, Becton Dickinson, Franklin Lakes, NY, USA), and resuspended in 195 *μ*L of 1× binding buffer (BB-10 mM Hepes/NaOH, pH 7.4, 140 mM NaCl, and 2.5 mM CaCl_2_). To the suspension, 5 *μ*L of fluorescein-conjugated Annexin V (Annexin V-FITC; Bender Med Systems, Wien, Austria) was added and samples were incubated for 10 min at room temperature. After washing, cells were resuspended in 190 *μ*L of BB in which 10 *μ*L of propidium iodide stock solution (final concentration 1 *μ*g/mL) was added. Flow cytometry analysis was done on CyAN, Dako Cytomation using the Summit software.

### 2.5. Quantitative RT-PCR

Total RNA from cell line, treated for 72 h with SAHA 1 *μ*M and PJ34 15 *μ*M alone or in combination, was extracted with RNeasy mini kit according to manufacturer's instructions (Qiagen, Hilden, Germany). 500 ng of total RNA was reversely transcripted to cDNA using random exaprimers and MMLV reverse transcriptase (Invitrogen). Real-time PCRs were performed using TaqMan Universal PCR Master Mix (Applied Biosystems) with the ABI Prism 7300 Sequence Detection Systems (Applied Biosystems, Foster City, CA, USA). The ΔΔCT method, by means of the SDS software (Applied Biosystems), was used to calculate the mRNA levels. Oligonucleotide primers were purchased from Life Technologies and were as follows: *β*-actin 5′ primer CGAGCGCGGCTACAGCTT, *β*-actin probe ACCACCACGGCCGAGCGG, and *β*-actin 3′ primer TCCTTAATGTCACGCACGATTT; PAX8 5′ primer CAACAGCACCCTGGACGAC, PAX8 3′ primer AGGGTGAGTGAGGATCTGCC, and PAX8 probe CTGACCCCTTCCAACACGCCACTG; NIS Hs00166567_m1; TTF1 Hs00968940_m1; TTF2 Hs00916085_s1; and TSHR Hs01053846_m1.

### 2.6. Statistical Analysis

Cell viability, apoptosis, and mRNA levels were expressed as means ± SD, and significances were analyzed with the* t* test performed with GraphPAD Software for Science (San Diego, CA, USA).

## 3. Results

**Figure 1:**
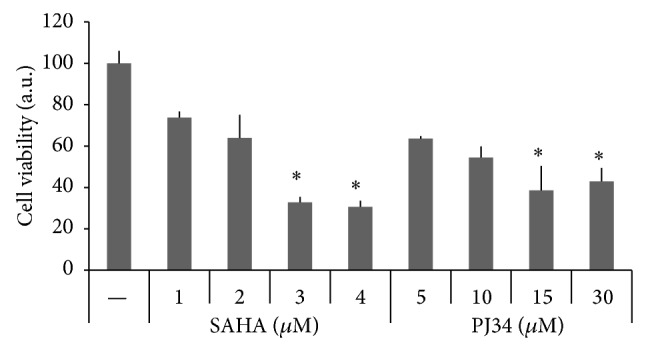
Effects of HDAC and PARP inhibitors on SW1736 cell viability. Cells were treated for 72 h with SAHA (1 *μ*M–4 *μ*M) or PJ34 (5 *μ*M–30 *μ*M), and CellTiter-Blue Cell Viability assay was performed as described in Section 2. Bars indicate the percentage of viable cells versus controls (untreated cells) and represent means ± SD of three experiments. ∗ indicates values significantly different compared to control.

In a first set of experiments, single effects of the HDAC inhibitor SAHA and the PARP inhibitor PJ34 on cell viability of the human anaplastic thyroid cancer-derived cell line SW1736 were investigated. Cell viability was assessed after treatment with different doses of SAHA and PJ34 for 72 hours (Figure 1). Both SAHA and PJ34 alone inhibited cell proliferation in a dose-dependent manner; however, at the utilized doses, SAHA seemed to have a greater effect, causing a more significant decrease in cell viability compared to cells treated by PJ34. Thus, both compounds alone were able to inhibit proliferation of SW1736 cells. We then tested synergy of the two compounds by measuring CI values of different drug combinations according to the Chou-Talalay equation [14, 15]. As indicated in Table 1, all combinations used showed a very high decrease in cell growth compared to untreated cells (always the CI values were lower than 1). Our results indicated that SAHA and PJ34 have a synergic effect in decreasing cell proliferation in a quite high range of utilized doses.

**Figure 2:**
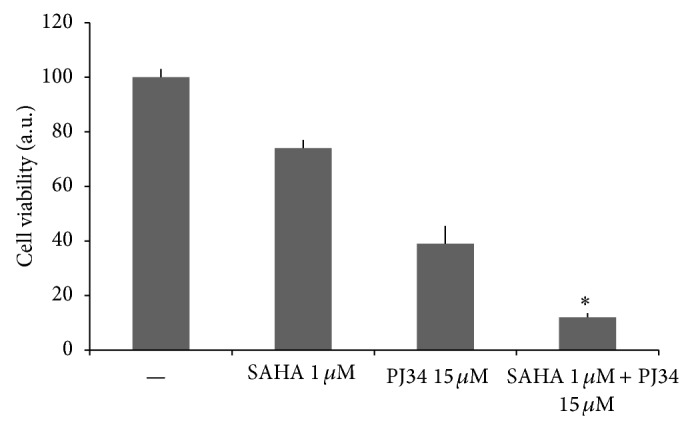
Effect of HDAC and PARP inhibitors combination on SW1736 cell viability. Cells were treated for 72 h with SAHA 1 *μ*M and PJ34 15 *μ*M, alone or in combination. CellTiter-Blue Cell Viability assay was performed as described in Section 2. Bars indicate the percentage of viable cells versus controls (untreated cells) and represent means ± SD of four experiments. ∗ indicates values significantly different compared to all other conditions.

We focused on the SAHA 1 *μ*M and PJ34 15 *μ*M combination which generated the lowest CI value. As represented in Figure 2, the treatment with SAHA 1 *μ*M had only a light effect on SW1736 viability, while PJ34 15 *μ*M reduced cell proliferation more effectively. Using these doses in combination we obtained a strong lowering of cell viability, almost 90% compared to untreated cells.

**Figure 3:**
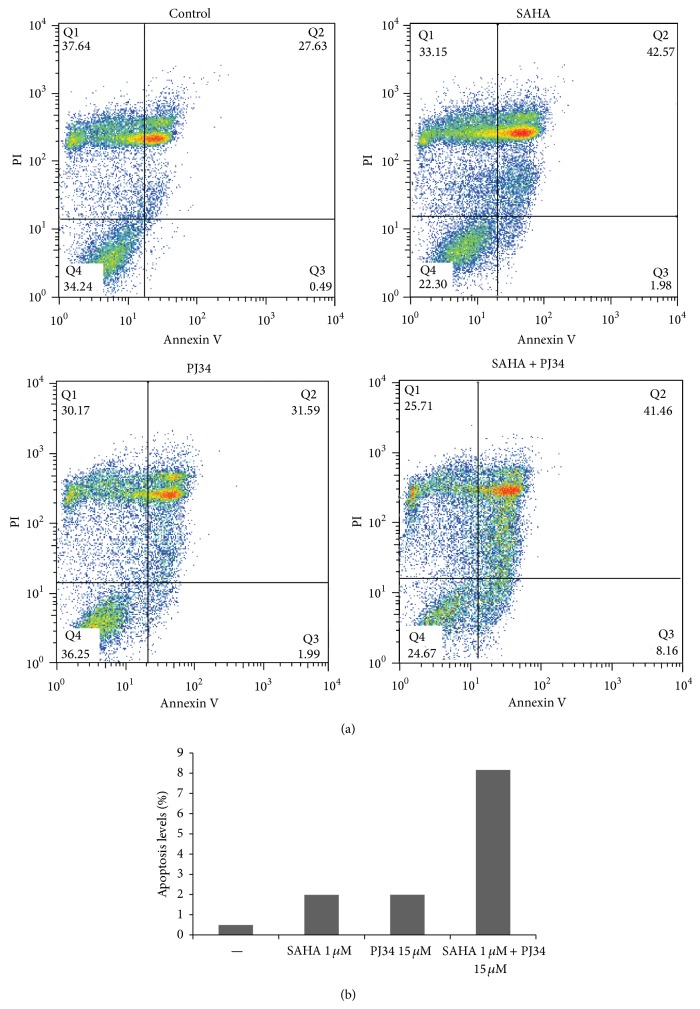
Combination of SAHA and PJ34 induces apoptosis in SW1736 cell line. In (a) Annexin V (FITC) and propidium iodide (PI) staining of anaplastic thyroid cancer cell line after 72 h treatment with SAHA 1 *μ*M, PJ34 15 *μ*M alone or in combination. In (b) representation of Annexin V-positive/PI-negative cells.

Subsequently, we evaluated apoptosis of SW1736 by measurement of Annexin V by fluorescence-activated cell sorting after treatments with SAHA 1 *μ*M and PJ34 15 *μ*M, alone or in combination (Figure 3(a)). The percentage of apoptotic cells (Annexin V-positive/PI-negative) was 1.98% with SAHA 1 *μ*M and 1.99% with PJ34 15 *μ*M while after the combination treatment, it reached 8.16% (Figure 3(b)).

**Figure 4:**
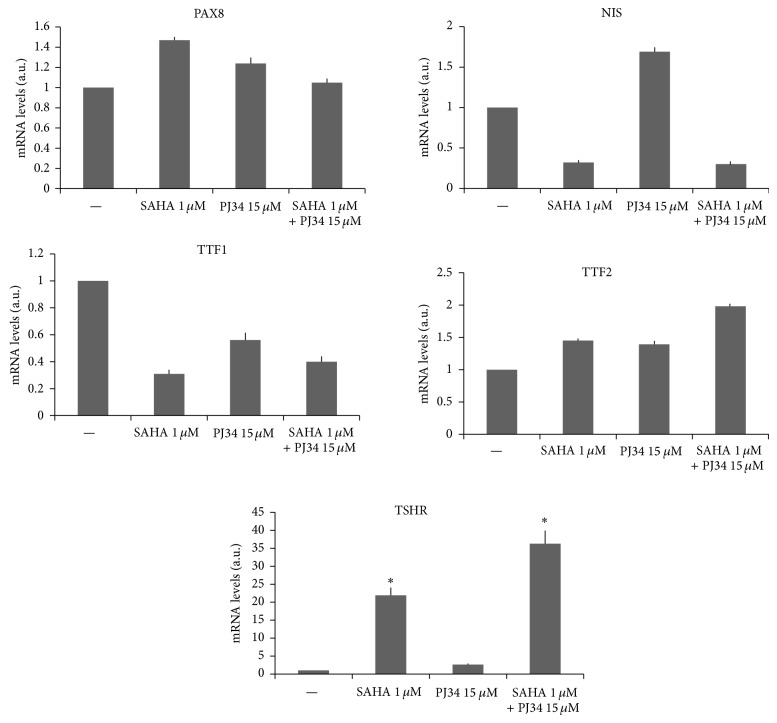
Expression levels of PAX8, NIS, TTF1, TTF2, and TSHR genes in SW1736 cell line. RNA extraction and real-time PCR are described in Section 2. For each gene the results were normalized against *β*-actin and expressed in arbitrary unit (2^−ΔCt^). Each bar represents the mean value of three different determinations. For each bar standard deviation is not above 10% of each value. ∗ indicates values significantly different compared to all other conditions.

We next tested if synergy between the two compounds was present on expression of several thyroid-specific genes. Effects on mRNA levels were evaluated by real-time PCR. Among all genes analyzed synergy between the two compounds was detectable only for TSHR gene (Figure 4). The 72-hour treatment with SAHA 1 *μ*M induced a marked effect on TSHR mRNA expression, while PJ34 15 *μ*M did not have any remarkable effect compared to the control cells. However, by using the two drugs in combination we obtained a strong effect on TSHR mRNA levels, significantly higher than all other conditions, with an increment of 36-fold of induction compared to the control.

## 4. Discussion

Developing a pharmacological treatment against cancer, the central issue consists in increasing therapeutic index and, at the same time, limiting development of resistance. One solution is to combine multiple drugs that act synergistically, and, in fact, a large number of ongoing clinical trials are investigating the effects of combined therapy against different types of cancer [16, 17]. In developing new strategies for treatment of anaplastic thyroid cancer, combinations of HDAC inhibitors and other drugs have been attempted [18–24]. However, neither in preclinical nor in clinical settings of thyroid cancer treatment, the combination between HDAC and PARP inhibitors has been investigated. It is increasingly clear that cancer is not only caused by genetic factors but can also be considered a epigenetics disease [25] and epigenetic enzymes can, therefore, be considered as novel therapeutic targets. Accordingly, both HDAC and PARP inhibitors can be considered as epigenetic drugs. Combinations of HDAC and PARP inhibitors have been tested in different kinds of neoplastic diseases. By using hepatocellular carcinoma cell lines, a synergistic inhibition of cell growth by SAHA and the PARP inhibitor olaparib has been demonstrated [26]. Inhibition of cell proliferation was associated with increase of apoptosis levels, accumulation of DNA damage, and modification of the cAMP signaling pathway. Combined effects of SAHA and PJ34 on leukemia cell lines have been investigated [27]. Also in that study, synergistic effects on proliferation inhibition and apoptosis increase have been observed. Recently, synergy between SAHA and olaparib has been observed even in ovarian cancer cell lines [28]. We have recently obtained similar effects on breast cancer cell lines (unpublished data).

Effects of HDAC and PARP inhibitors alone have been previously investigated by our group. We have shown that HDAC inhibitors affect cell proliferation and expression of various genes in several thyroid cancer cell lines [29, 30]. Moreover Lavarone et al. have recently shown that PJ34 inhibits cell growth and increases NIS expression in various thyroid cancer cell lines [31]. In this research we demonstrate that HDAC and PARP inhibitors have a synergistic effect on proliferation of a human anaplastic thyroid-derived cell line. Thus, synergy between these two classes of compounds appears to be a common phenomenon in cancer cell lines of various origins, underlying the potential role of these combinations as an interesting strategy for cancer therapy. Our obtained CI values indicate that synergy between SAHA and PJ34 occurs in a wide range of doses, suggesting that the combined effect could probably be observed also* in vivo*.

In addition to the impact on cell proliferation, we have investigated effects of the SAHA-PJ34 combination on thyroid-specific genes expression. Regarding thyroid-specific transcription factors, the SAHA-PJ34 combination induces a TTF1 slight decrease, a TTF2 slight increase, and no change in PAX8. Such behavior indicates that the control of these genes expression occurs through distinct mechanisms. This view agrees with previous studies in which control of thyroid-specific transcription factors expression has been investigated [32].

Synergy between SAHA and PJ34 in increasing mRNA levels of TSHR was observed. The TSHR is localized in the plasma membrane, and, thus, it has been proposed as a target to direct therapeutic compounds into thyroid cancer cells [33–36]. Our data would suggest that the combined use of HDAC and PARP inhibitors may facilitate such approach.

Differently from TSHR, NIS gene expression is reduced by SAHA alone and in combination with PJ34. Such a different effect between these genes expression is not unexpected. TSHR and NIS have a different regulation in terms of gene expression. It is well known, for example, that during tumorigenesis NIS is one of the earliest downregulated genes, while TSHR is among the latest ones [37, 38].

In conclusion, considering our data on cell proliferation and gene expression altogether, the combined use of HDAC and PARP inhibitors can be a useful strategy for ATC treatment. Preclinical* in vivo* studies are required to validate such a possibility.

## Figures and Tables

**Table 1:** Combination index data for SAHA and PJ34 combination.

Dose SAHA (*μ*M)	Dose PJ34 (*μ*M)	Combination effect (% cell viability)^*^	CI value
1.0	5.0	26	0.26979
1.0	10.0	37	0.60235
1.0	15.0	12	0.13878
1.0	30.0	13	0.18473
2.0	5.0	18	0.33123
2.0	10.0	17	0.33414
2.0	15.0	19	0.40111
2.0	30.0	11	0.25341
3.0	5.0	13	0.36504
3.0	10.0	26	0.75320
3.0	15.0	16	0.47175
3.0	30.0	14	0.45413
4.0	5.0	17	0.60763
4.0	10.0	17	0.62785
4.0	15.0	10	0.40318
4.0	30.0	13	0.53865

^*^Mean value of four replicates. In each condition standard deviation is less than 10%.
